# Predicting compressive strength of high-performance concrete with high volume ground granulated blast-furnace slag replacement using boosting machine learning algorithms

**DOI:** 10.1038/s41598-022-12890-2

**Published:** 2022-06-09

**Authors:** Vimal Rathakrishnan, Salmia Bt. Beddu, Ali Najah Ahmed

**Affiliations:** 1 Department of Civil Engineering, College of Engineering, Universiti Tenaga Nasional (UNITEN), 43000 Selangor, Malaysia; 2 Institute of Energy Infrastructure (IEI), Universiti Tenaga Nasional (UNITEN), 43000 Selangor, Malaysia

**Keywords:** Civil engineering, Mechanical properties

## Abstract

Predicting the compressive strength of concrete is a complicated process due to the heterogeneous mixture of concrete and high variable materials. Researchers have predicted the compressive strength of concrete for various mixes using machine learning and deep learning models. In this research, compressive strength of high-performance concrete with high volume ground granulated blast-furnace slag replacement is predicted using boosting machine learning (BML) algorithms, namely, Light Gradient Boosting Machine, CatBoost Regressor, Gradient Boosting Regressor (GBR), Adaboost Regressor, and Extreme Gradient Boosting. In these studies, the BML model’s performance is evaluated based on prediction accuracy and prediction error rates, i.e., R^2^, MSE, RMSE, MAE, RMSLE, and MAPE. Additionally, the BML models were further optimised with Random Search algorithms and compared to BML models with default hyperparameters. Comparing all 5 BML models, the GBR model shows the highest prediction accuracy with R^2^ of 0.96 and lowest model error with MAE and RMSE of 2.73 and 3.40, respectively for test dataset. In conclusion, the GBR model are the best performing BML for predicting the compressive strength of concrete with the highest prediction accuracy, and lowest modelling error.

## Introduction

### Literature review and problem statement

Concrete has been commonly used in construction and architecture due to its favourable engineering properties. Concrete has the characteristics of rich raw material, low price, and high compressive strength and good durability^1^. Concrete comprises four primary components: coarse aggregate, fine aggregate, cement, and water. Concrete's economic value allows it to be widely used in constructions and the accessibility to the material available in the local market. It also demonstrates excellent benefits over other construction materials such as steel, and concrete can be produced with minimum effort. In certain instances, supplementary materials like fly ash (PFA)^2,3^, blast furnace slag (GGBS)^4^, silica fume^5^, and other industrial waste/by-products are added in concrete to enhance the mechanical properties of the concrete^4^. The introduction of industrial waste/by-product^6,7^ into concrete offers environmental benefits while increasing the longevity and resiliency of concrete structures.

Among the various concrete property indices, compressive strength is the most critical because it is directly related to the structural safety and is required for determining the performance of structures throughout their life, from new structural design to old structural assessment^8^.

When dealing with concrete materials, one of the difficulties in selecting the appropriate materials and predicting the mechanical properties of the concrete, i.e., compressive strength, is due to cost and the availability of local material^9^. It is vital to have robust and reliable predictive models based on existing input and output data at the early stage to drive down the cost of making further experiments and reduce the cost associated with the risk of non-compliance concrete during construction^5^. With the use of suitable models, it can lead to success in finding combination inputs that can achieve meaningful outcomes and, at the same time, saves considerable time and money. However, empirical, and statistical models, such as linear and nonlinear regression, have been widely used. However, these models require laborious experimental work to develop, and can provide inaccurate results when the relationships between concrete properties and mixture composition and curing conditions are complex^10^.

**Figure 1:**
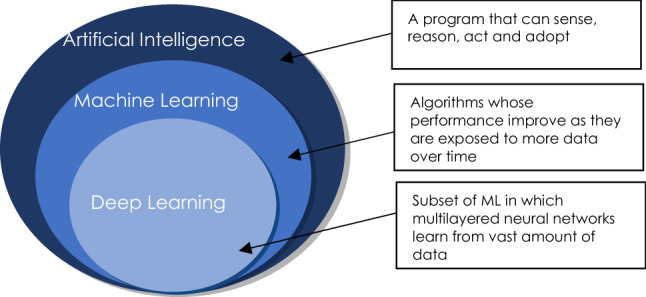
Artificial Intelligence sub-classes.

ML is a sub-class of AI that self-learning through algorithms and improves its performance based on previous datasets/experience. The distinction between AI, ML, and DL is illustrated in Fig. 1. With minimal human input, ML algorithms will automatically learn and improve over time^6^. ML has been widely applied in the field of engineering to solve a variety of problems i.e., predict outages, estimate angular velocity, components failure prognostics and prediction of fatigue life^11–14^. In civil engineering, AI and ML have been previously employed to tackle problems in various structural engineering fields^15^. ML application is also used in building structural design & performance assessment, improving finite element modelling of structures, and enhancing concrete properties prediction & assessment^9,16–20^.

Given the popularity of machine learning, especially in concrete technology, various studies have been conducted using ML/DL approaches^10^. Table 1 below shows the summary of concrete compressive strength prediction for various types of concrete using various ML and DL models. Many empirical and statistical models, i.e., linear and nonlinear regression algorithms, were employed to predict the properties of concrete^10^. Multiple Linear Regression (MLR)^21^, Support Vector Machine (SVR)^22,23^, Multilayer Perceptron (MLP)^24^, and Gradient Boosting^25,26^ are most used ML algorithms to predict the mechanical and chemical properties of concrete. In general, the compressive strength prediction was undertaken for several type of concrete i.e., ordinary concrete^8,10^, high-performance concrete^25,27–30^, ultra-high-performance concrete ^20^, and green concrete with supplementary cementitious material i.e., fly ash^16,31,32^, blast furnace slag^4^ and recycled aggregates^6^. ML/DL is also used to predict other mechanical and chemical properties of concrete, i.e., prediction of concrete shear strength^15,24^, 30], tensile strength^33^, flexural strength^5^, the thermal conductivity of concrete^34^, and chloride concentration of concrete^35^.

**Table 1:** Summary of previous studies on concrete strength prediction.

No	Type of Concrete	Model	Dataset	Year	Reference
1	Fly-ash based concrete	Decision tree, ensemble bagging, Gene expression programming	270	2021	^32^
2	High-performance concrete from industrial wastes	Decision tree, random forest, support vector, artificial neural network, multiple linear regression, ensemble bagging & boosting	1030	2021	^43^
3	Self-compacting concrete with fly-ash	Data Envelopment Analysis	114	2021	^44^
4	Steel fibre-reinforced concrete	Boosting- and tree-based models, K-nearest neighbour, linear, ridge, lasso regressor, support vector regressor, multilayer perceptron models	220	2021	^5^
5	Self-compacting concrete with high-volume fly ash	Support vector machine	337	2020	^23^
6	High-performance concrete	Multivariate adaptive regression splines, kernel ridge regression, gradient boosting machines, gaussian process regression	1030	2020	^25^
7	High-strength concrete	Gene expression programming	357	2020	^27^
8	Ultra-high-performance concrete	Artificial neural network: Sequential Feature Selection (SFS) and Neural Interpretation Diagram (NID)	110	2020	^20^
9	Alkali-activated concrete	Random Forest	180	2020	^3^
10	Ordinary concrete	Extreme gradient boosting	1030	2020	^45^
11	Self-compacting concrete	Artificial neural network	205	2019	^46^
12	Self-compacting concrete with fly ash	Enhanced multiclass support vector machine and fuzzy rule	114	2019	^16^
13	Lightweight self-compacting concrete	Random forest regression	131	2019	^47^
14	High-performance concrete	Artificial neural network: modified firefly algorithm	1133	2018	^33^
15	High-performance concrete	Support vector machine, enhanced cat swarm optimisation	2200	2018	^48^
16	Lightweight Aggregate Concretes	Extreme learning machine regressor, particle swarm optimization	75	2018	^49^
17	Self-compacting concrete containing fly ash	Decision tree algorithms: M5′ and multivariate adaptive regression splines	114	2018	^31^

For the DL model, Artificial Neural Network (ANN)^4,7,29,36–38^ was widely used in most previously reported studies. The use of boosting algorithms is not extensively reported in any previous studies except the GBR models. The proposed boosting algorithms were chosen based on their popularity and frequency in other research areas such as biomedical and construction hazard analysis, which reports that the BML models have higher prediction accuracy than other ML and DL models^39–42^. We implemented and analysed the accuracy and error of compressive strength prediction for five different boosting algorithms, namely LBGM, CATB, GBR, ADAB, and XGB. Additionally, the BML models are enhanced using the Random Search (RS) optimization process, which involves tuning the hyper-parameters of the BML algorithms.

### Objectives

The study's objective is to identify the best performing BML models, i.e., LBGM, CATB, GBR, ADAB, and XGB to predict the HPC with high volume GGBS using BML algorithms, i.e., LBGM, CATB, GBR, ADAB, and XGB. The BML models were then optimised using the Random Search (RS) optimisation process by tuning the hyper-parameters of each BML model function. Additionally, comparison studies were also conducted using commonly used ML models, i.e., linear regression, decision tree, random forest, etc., to evaluate the performance of the BML model in predicting the concrete strength.

The fundamentals behind BML algorithms models are defined in Sect. 2, followed by the statistical properties analysis of the dataset & modelling approach, findings from the optimised BML model, comparison studies between other ML models, and model validation results are provided in Sect. 3. The findings of each model’s prediction accuracy and modelling errors are concluded in Sect. 4.

## Methodology

### BML algorithms

#### Light gradient boosting machine (LBGM)

LGBM is a gradient boosting framework that uses tree-based learning algorithms developed by Microsoft^50^. LBGM uses two innovative sampling techniques: Gradient-based One-Side Sampling (GOSS) and Exclusive Feature Bundling (EFB). GOSS excludes a substantial fraction of data instances with small gradients and uses the remainder to estimate the information gain. Since data instances with large gradients contribute more to the computation of information gain, GOSS can generate a highly accurate estimate of information gain with a significantly smaller data set.

EFB allows for the grouping of mutually exclusive features, hence reducing the number of features. It also demonstrates that while determining the optimal bundling of exclusive features, a greedy approach can reach an approximation ratio of relatively high. It was reported that LGBM speeds up the training process of conventional GBDT by up to over 20 times while achieving almost the same accuracy, and it is six times faster than XGBoost^50^.

#### CAT boost regressor (CATB)

CATB is an open-sourced machine learning algorithm developed by Yandex in 2017. CATB is a decision tree algorithm based on gradient boosted decision trees. The algorithms in CATB models are a series of decision trees constructed sequentially, with each new tree having a lower loss than the prior trees. The starting parameters determine the number of trees generated, and overfitting is avoided using an overfitting detector. The processes of tree construction for a single tree in CATB algorithms include computing splits in advance, converting categorical features to numerical features, selecting the tree structure, and calculating values in leaves.

Generally, CATB employed greedy algorithms in optimising the prediction accuracy. The features of CATB models are ordered according to their splits and are then substituted in each leaf. The depth of the tree and other constraints for structure selection is specified with pre-modeling parameters, and a random permutation of classification/regression objects is conducted before the construction of each new tree. CATB models validate the model performance with a metric that indicates the direction in which the function should be improved further when deciding the construction of the next tree. CATB model surpasses leading GBR packages and achieves new state-of-the-art performance on common benchmarks^51,52^.

#### Gradient boosting regressor (GBR)

Friedman presented the GBR model as an ensemble method for regression and classification in 1999. The gradient boosting approach compares each iteration of the randomly chosen training set to the base model. In the GBR model, the lower the training data fraction, the faster the regression, as the model fits smaller data each iteration. GBR model requires the following tuning parameters: ntrees and shrinkage rate, where ntrees is the number of trees to be grown, and the shrinkage parameter, often referred to as the learning rate applied to each tree in the expansion^25,53^.

This algorithm's fundamental foundation is 'boosting.' The boosting process aids in transitioning prediction from a 'weak' learner via the additive training process. The essential advantage of GBR algorithms is that it avoids overfitting and makes efficient use of computational resources by using an objective function. Besides improving output performance, GBR algorithms reduce the selected error function further^54^.

#### Adaboost regressor (ADAB)

ADAB, an acronym for Adaptive Boosting, is a meta-algorithm for statistical categorization developed in 2003 by Yoav Freund and Robert Schapire. It can be combined with a variety of other types of learning algorithms to enhance performance. The output of the other learning algorithms, i.e., 'weak learners,' is combined into a weighted sum representing the boosted classifier's final output. ADAB is adaptive because it adjusts succeeding weak learners favoring instances misclassified by previous classifiers/regressors. It is less prone to overfitting than other learning algorithms in some cases^29^.

The individual learners in ADAB algorithms may be ineffective. Still, if their performance is marginally better than random guessing, the final model can be demonstrated to converge to a powerful learner. This technique benefits from a single best-fit decision model formed from the outcomes of several decision trees, each of which is constructed using a random selection of base features, i.e., decision factors from a training dataset^55,56^.

#### Extreme gradient boosting (XGB)

**Figure 2:**
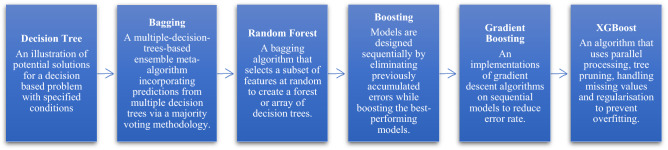
The evolution of XGBoost.

Extreme Gradient Boosting (XGB) or XGBoost is a decision tree-based ensemble ML algorithm that uses gradient boosting to make predictions for unstructured data. Tianqi Chen and Guestrin developed XGBoost, and the method uses the conventional tree gradient boosting algorithm^45^ to create state-of-the-art algorithms, the ‘extreme gradient boosting’^23^. The multiple Kaggle competition winner ‘XGBoost’ is a highly effective ML algorithm due to its scalable tree boosting system and sparsity-aware algorithm in modelling structured datasets. The algorithm has been the source of countless cutting-edge applications, and it has been the driving force behind many of these recent advances. It’s been widely used as industrial solutions such as customer churn prediction^57^, applicant risk assessment^58^, malware detection^59^, stock market selection^60^, classification of traffic accidents^61^, diseases identification^40^, and even in predicting the death of patience during SARS-COV-2(Covid-19) treatment^42^. The most significant benefit of XGBoost is its scalability across any condition^62^. In general, the XGBoost algorithms are the evolution of decision tree algorithms that were improved over time. Figure 2 below shows the development of decision tree-based algorithms to XGBoost.

### Model structure

**Figure 3:**
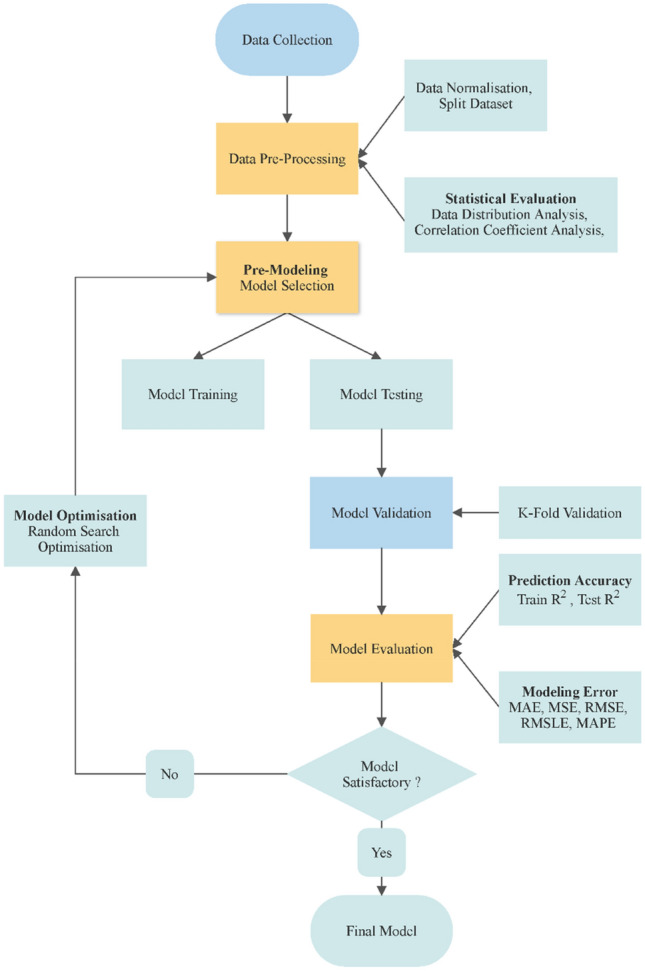
Step by step BML modelling approach.

For the most part, we utilised the Python programming language on Google's Colab platform to analyse the data and create the models. An open-source, low-code machine learning library ‘PyCaret’ was used in research^63^. Figure 3 illustrates the step-by-step procedure for training, optimising, and validating the BML models in predicting the concrete compressive strength. Seven key processes are involved in the development of the optimised BML model, and each stage is described in detail below, with brief explanations:

i. Data Collection – This entails collecting data from the laboratory and compiling it appropriately.
ii. Data Pre-Processing – To correctly identify and arrange the acquired data, it is necessary to sort out the missing values and then normalise the dataset in preparation for model building.
iii. Model Selection—For prediction and evaluation in this research, BML algorithms, i.e., LBGM, CATB, GBR, ADAB, and XGB, were utilised.
iv. Hyper-parameter Optimisation – The RS approach was employed in each of the five proposed BML algorithms, and the results are compared to the original models.
v. Model Validation—Validation and testing of the models were performed using the k-fold cross-validation approach, which randomly splits the dataset and minimises overfitting.
vi. Model Evaluation – All the models are compared, and the best performing algorithms are selected based on evaluation metrics, i.e., R^2^, RMSE, MAE, MSE, RMSLE, MAPE.
vii. Analysis and Reporting – The findings in the case study are reported based on comparing various ML models, optimisation parameters, and evaluation metrics.

### Data collection and pre-processing

#### Overview

A total of 152 data of HPC compressive strength data were gathered from concrete trial mix conducted at a laboratory in Selangor, Malaysia. In general, the dataset is composed of seven concrete components: fine aggregate, coarse aggregate, ordinary Portland cement (OPC), ground granulated blast-furnace slag (GGBS), silica fume (SF), water, admixture, and moisture content (MC). The dataset also contains concrete compressive strength of a Grade 80 HPC, and the compressive strength results are available for 7, 28, 56, and 91 days. On average, each batch of concrete contains around 246 kg of GGBS and OPC, respectively.

The proportion of cementitious content in each batch is around 45% of GGBS, 45% of OPC, and 10% of SF. Similarly, the ratio of fine to coarse aggregate is 1:1, equating to 70% of total concrete volume with a 0.25 water-to-cement ratio, or 138 kg of water in each batch of concrete. Additionally, the moisture content of fine and coarse aggregate was included as input parameters since the water content in each concrete batches was adjusted according to the moisture content in the aggregates. Details of statistical metrics are listed in Table 2 below.

**Table 2:** Summary of statistical analysis of the concrete material composition.

	Fine Agg	Coarse Agg	GGBS	OPC	SF	Water	Admixture	Fine MC	Coarse MC	Days	Strength
count	152	152	152	152	152	152	152	152	152	152	152
mean	871.7	874.3	246.6	246.3	54.4	138.7	12.2	4.4	0.5	45.5	105.2
std	11.7	10.2	1.4	0.9	0.6	2.9	0.5	0.7	0.2	31.6	14.6
min	842.0	857.0	244.0	244.0	53.0	135.0	11.5	3.2	0.2	7.0	70.3
max	900.0	904.0	250.0	248.0	56.0	149.0	12.7	6.0	1.0	91.0	131.4

#### Data distribution analysis

**Figure 4:**
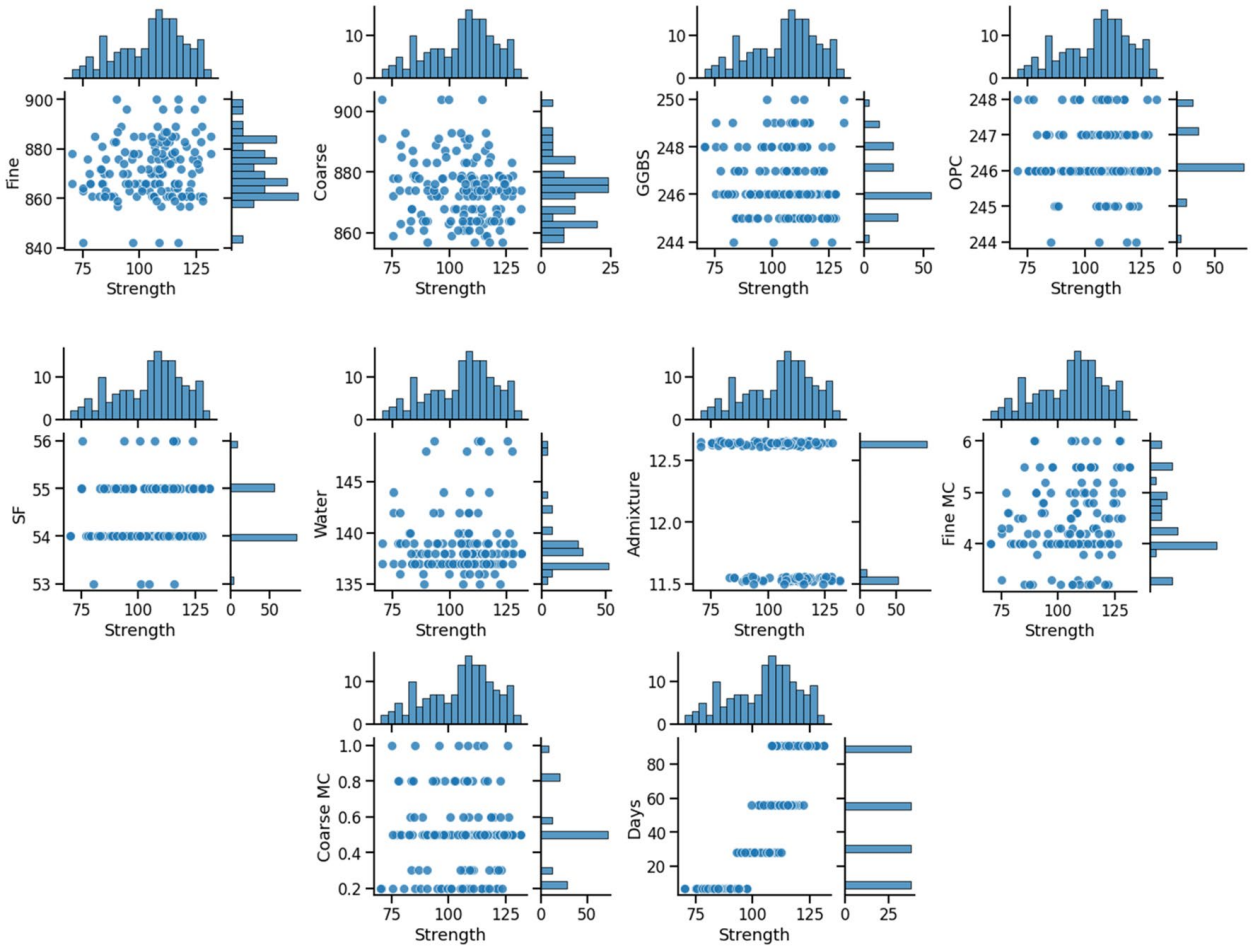
Distribution correlation of input parameters and strength.

The distribution correlations between the input parameters and the compressive strength are shown in Fig. 4. It illustrates the correlation between the data points by including the relative frequency distribution of each input parameter. Generally, the distribution of input parameters suggests that the dataset is appropriately distributed and fit for machine learning modelling.

#### Correlation coefficient analysis

**Figure 5:**
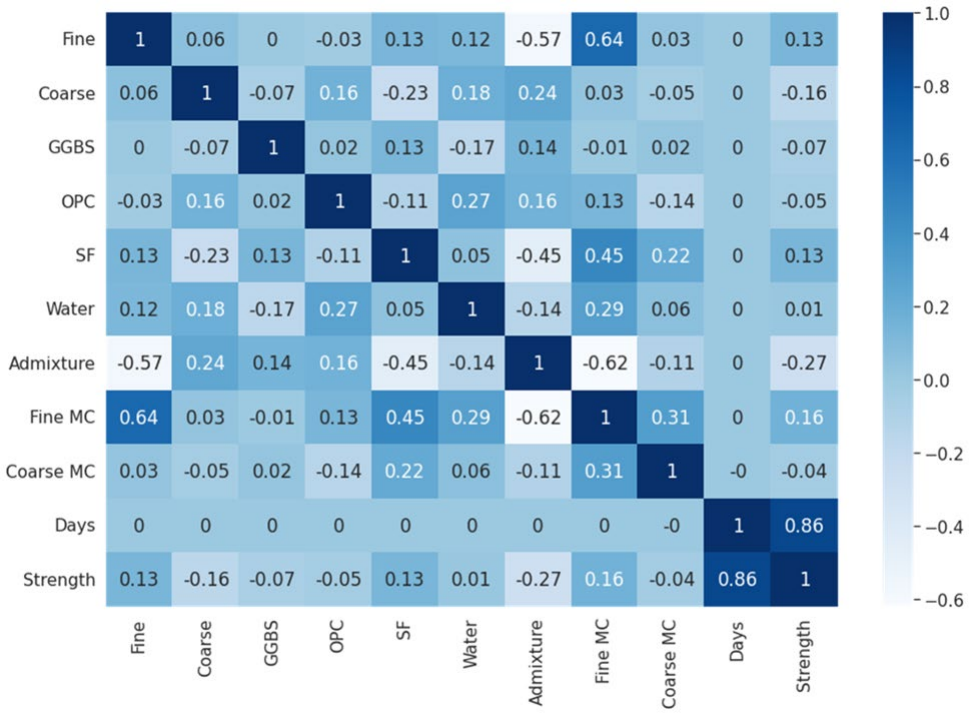
Pearson’s correlation heatmap.

Along with statistical and distribution analysis, a correlation coefficient study was performed to analyse the dataset and prepare for modelling. Pearson’s correlation coefficient approach indicated in Eq. 1 was used to calculate the correlation coefficient^64^. Pearson's correlation coefficient is a test statistic that shows the statistical link between two continuous variables. It is based on the covariance approach, in which the best method is considered for determining the relationship between two variables of interest. It reveals both the size of the association or correlation and the direction of the relationship. The correlation between all parameters was analysed for this research and visualized in Fig. 5 as a Pearson’s correlation heatmap.

1 $$Pearson\, Correlation\, Coefficeint, r = \frac{{\sum \left( {x_{i} - \overline{x}} \right)\left( {y_{i} - \overline{y}} \right)}}{{\sqrt {\sum \left( {x_{i} - \overline{x}} \right)^{2} \sum \left( {y_{i} - \overline{y}} \right)^{2} } }}$$

where;

$$r$$ = correlation coefficient.

$x_{i}$  = values of the x-variable in a sample.

$\overline{x}$ = mean of the values of the x-variable.

$y_{i}$ = values of the y-variable in a sample.

$\overline{y}$ = mean of the values of the y-variable.

As shown in Fig. 5 above, it can be observed that the correlation between input and output parameters is relatively low and generally in the range of -0.62 to 0.64. The range of the correlation coefficients indicates that the input variables can be considered low to moderately correlated to the compressive strength.

#### Data split and normalisation

The dataset's modelling proportion was randomly partitioned into two sets, i.e., training and testing dataset. Around 70% of the dataset was utilized for training the BML models, whereas 30% were used to test the models^65^, ^65^ Before training BML models, pre-processing data is required. To prevent training from being dominated by one or a few features with large magnitude, features should be normalised so that their range is consistent. The Z-score normalisation method was used in this study to normalise all values in a dataset so that the mean of all values is 0 and the standard deviation is 1. *Equation **2* below shows the formula to perform a z-score normalization on every value in a dataset:

2 $${\text{Z}} - {\text{Score}} = \left( {{\text{x }}{-} \, \mu } \right)/\sigma$$

where:

x: Original value.

μ: Mean of data.

σ: Standard deviation of dataset.

#### ***Model ***validation*** using K-Fold cross-validation***

**Figure 6:**
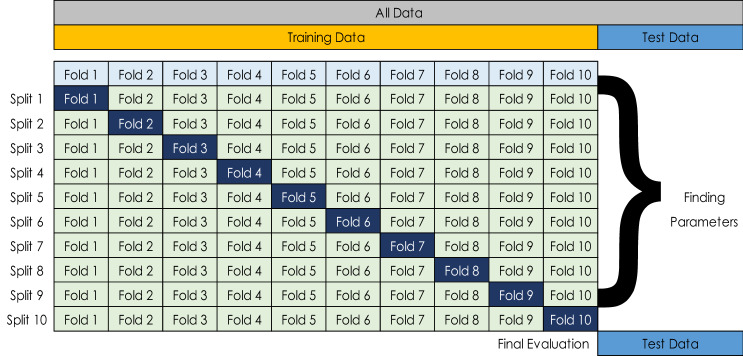
K-fold cross validation method.

Validation and testing of the models were performed using the k-fold cross-validation method illustrated in Fig. 6. In this study, a total of ten folds or k value of 10 were used. The dataset is randomly separated into test and training data and divided into k groups, using this cross-validation procedure. Validation of the model is performed on one of the groups, and training is performed on the remaining groups. The process is performed k times more until each distinct group is used as the validation set. The ultimate performance of the model is determined using test data that the model ‘not seen’ during training.

K-fold cross-validation enables the model to be trained and verified several times, resulting in a more accurate model with less overfitting. With the more traditional hold-out strategy, the dataset is partitioned into training, validation, and test sets, which reduces number of samples for model training. The model's performance is contingent upon a random selection of samples for the training, validation, and test sets.

#### ***Model ***evaluation

In this paper, six separate statistical measurement parameters were used to calculate the prediction efficiency of the BML models. In simpler terms, the evaluation parameters estimate the accumulated error in predictions concerning actual observations. The statistical parameters are: coefficient of determination (R^2^), mean absolute error (MAE), root mean squared error (RMSE), mean squared error (MSE), root mean squared logarithmic error (RMSLE), and mean absolute percentage error (MAPE). These mathematical formulations are defined in Eqs. 3–8; in this case, n is the total number of test dataset records while y′ and y are the predicted and measured values, respectively. The values of R^2^ would range from 0 to 1 – the closer the value is to 1, the higher fitting optimisation of the model is. The values MAE, RMSE, MSE, RMSLE, and MAPE are used to evaluate modelling error—the smaller the value, the lesser the difference between the predicted and measured values.

3 $$R^{2} = 1 - \frac{{\mathop \sum \nolimits_{i = 1}^{n} (\widehat{{y_{i} }} - y_{i} )^{2} }}{{\mathop \sum \nolimits_{i = 1}^{n} \left( {y_{i} - \overline{y}_{i} } \right)^{2} }}$$

4 $$MAE = \frac{1}{n} \mathop \sum \limits_{i = 1}^{n} \left| {y - y^{\prime}} \right|$$

5 $$RMSE = \sqrt {\frac{1}{n} \mathop \sum \limits_{i = 1}^{n} \left| {y - y^{\prime}} \right|^{2} }$$

6 $$MSE = \frac{1}{n} \mathop \sum \limits_{i = 1}^{n} \left| {y - y^{\prime}} \right|^{2}$$

7 $$RMSLE = \sqrt {\frac{1}{n} \mathop \sum \limits_{i = 1}^{n} (\log \left( {y_{1} + 1} \right) - log\left( {y^{\prime} + 1} \right)^{2} }$$

8 $$MAPE = \frac{1}{n} \mathop \sum \limits_{i = 1}^{n} \left| {\frac{{y - y^{\prime}}}{y}} \right|$$

## Results and discussion

### Initial modelling

The LBGM, CATB, GBR, ADAB, and XGB algorithms were initially modelled using their default hyper-parameter settings. Each model's performance is measured in terms of prediction accuracy and error rates, i.e., R^2^, MSE, RMSE, MAE, RMSLE, and MAPE. The findings of the initial modelling are summarised in Table 3 below.

**Table 3:** Summary of initial modelling.

	Model	R^2^	MAE	MSE	RMSE	RMSLE	MAPE
Training Dataset	LBGM	0.86	3.60	14.92	3.86	0.03	0.03
	CATB	0.85	3.61	21.80	4.67	0.05	0.04
	GBR	0.83	4.02	22.32	4.72	0.05	0.04
	ADAB	0.81	4.20	26.59	5.16	0.05	0.04
	XGB	0.81	3.95	26.87	5.18	0.05	0.04
Test Dataset	LBGM	0.94	3.29	16.80	4.10	0.04	0.03
	CATB	0.89	3.97	29.10	5.39	0.06	0.04
	GBR	0.93	3.24	17.82	4.22	0.05	0.03
	ADAB	0.89	4.17	28.46	5.33	0.06	0.04
	XGB	0.92	3.64	21.86	4.68	0.05	0.04

**Figure 7:**
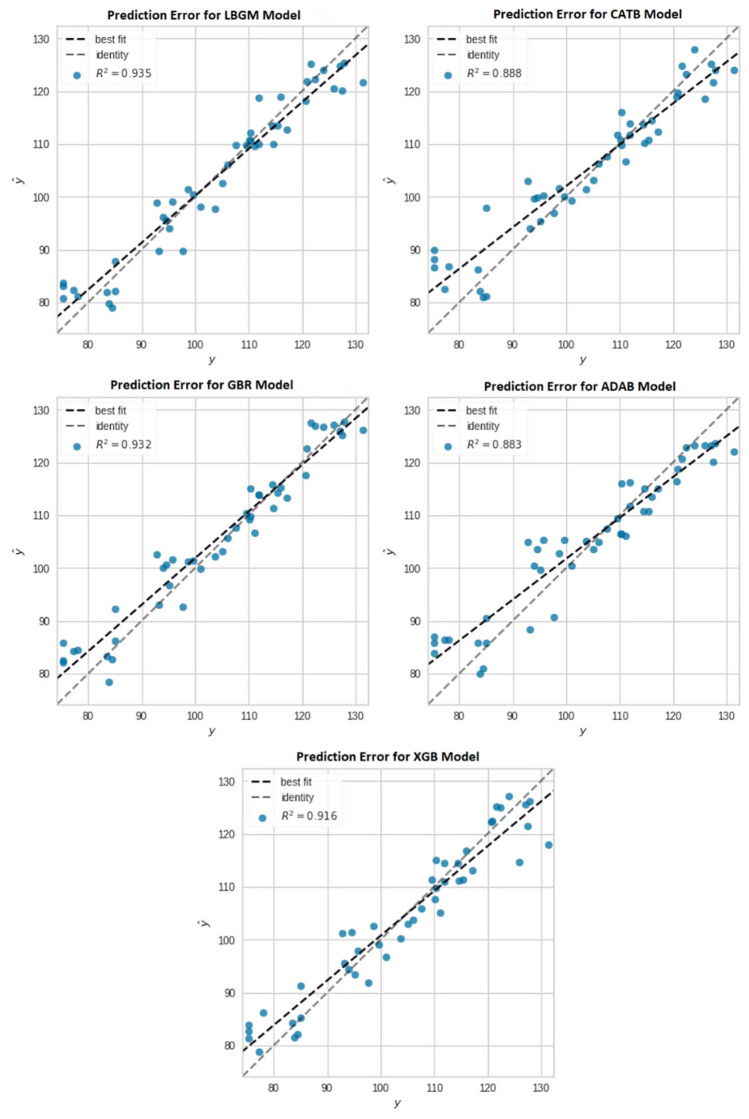
Best fit line for prediction distribution (RS model).

LBGM predicted the compressive strength of concrete with the highest prediction accuracy and the least prediction errors of all five BML models. The initial modelling of LBMG reached 0.86 and 0.94 for the training and testing prediction scores, respectively. GBR and XGB models also performed well, with prediction accuracy of 0.93 and 0.92 on the test dataset. The evaluation metrics in the LGBM model was the lowest in comparison to other BML models, with an MAE of 3.29, an RMSE of 4.10, and an RMSLE of 0.03 for test dataset. The GBR model was the second-best model in prediction errors with MAE and RMSE values of 3.24 and 4.22, respectively. The distribution of predicted results against actual results for all BML models are visualized in Fig. 7, along with the best fit line for the prediction distribution. The initial modelling suggests a reasonable prediction result; however, it is further improved by using the RS algorithm, which is discussed in detail in the following section.

In terms of prediction distribution, the LBGM and CATB models have the highest training scores of 0.86 and 0.85, respectively. In contrast, their prediction scores for the test dataset are significantly higher at 0.94 and 0.93. It suggests that both models concentrated on optimising the test score to get the maximum possible prediction accuracy. The prediction distribution indicates that the LBGM, GBR, and XBG results are closely spaced along the best fit lines compared to the CATB and ADAB models.

### Model optimisation with RS algorithm

The RS algorithm focuses on the use of random combinations to optimise the hyperparameters of a model. It measures random combinations of a set of values to optimise decent outcomes, with the function tested at any number of random combinations in the parameter space. The chances of discovering the optimal parameter are relatively higher in RS algorithms compared to Grid Search algorithm due to various search patterns in the model being trained on the optimised parameters without aliasing. RS algorithms are best for lower dimensional data as this method takes less time and iterations to find the right parameter combination^67^. Numerous hyperparameters were optimised in this study, including n_estimator, learning_rate, max_depth, and subsample and min_sample_split. A total of 1000 iteration was performed to identify the performing model and the optimum hyperparameters for each BML models. Table 4 below shows the hyperparameters and values used for all the model, before and after optimisation process.

**Table 4:** Summary of hyperparameter tuned values.

	Model	LGBM	CATB	GBR	ADAB	XGB
Default Value	n_estimator	100	100	1000	50	100
	learning_rate	0.10	0.10	0.03	1.00	0.03
	max_depth	−1	3	6	–	6
	subsample	1.00	1.00	0.80	–	1.00
Optimised Value	n_estimator	270	90	210	290	100
	learning_rate	0.20	0.30	0.15	0.40	0.30
	max_depth	−1	2	2	–	6
	subsample	1.00	0.80	0.65	–	1.00

Based on the optimised BML models, the GBR model achieved the highest prediction accuracy of 0.96 for the test dataset followed with LBGM and CATB model with R^2^ of 0.95. In comparison, the optimised GBR model had the lowest prediction errors for test errors, with an MAE of 2.73, an RMSE of 3.40, respectively. For training dataset, the CATB model recorded lowest prediction error and highest prediction accuracy of 0.89. Table 5 shows the summary of prediction accuracy and evaluation metrics for the optimised BML models.

**Table 5:** Summary of RS optimised models.

	Model	R^2^	MAE	MSE	RMSE	RMSLE	MAPE
Training Dataset	LBGM	0.88	3.27	16.22	4.03	0.04	0.03
	CATB	0.89	3.15	14.85	3.85	0.04	0.03
	GBR	0.88	3.26	16.75	4.09	0.04	0.03
	ADAB	0.83	4.00	24.50	4.95	0.05	0.04
	XGB	0.88	3.23	16.50	4.06	0.04	0.03
Test Dataset	LBGM	0.95	2.88	12.79	3.58	0.04	0.03
	CATB	0.95	2.98	13.30	3.65	0.04	0.03
	GBR	0.96	2.73	11.53	3.40	0.03	0.03
	ADAB	0.90	3.98	26.11	5.11	0.05	0.04
	XGB	0.94	3.14	15.20	3.90	0.04	0.03

**Figure 8:**
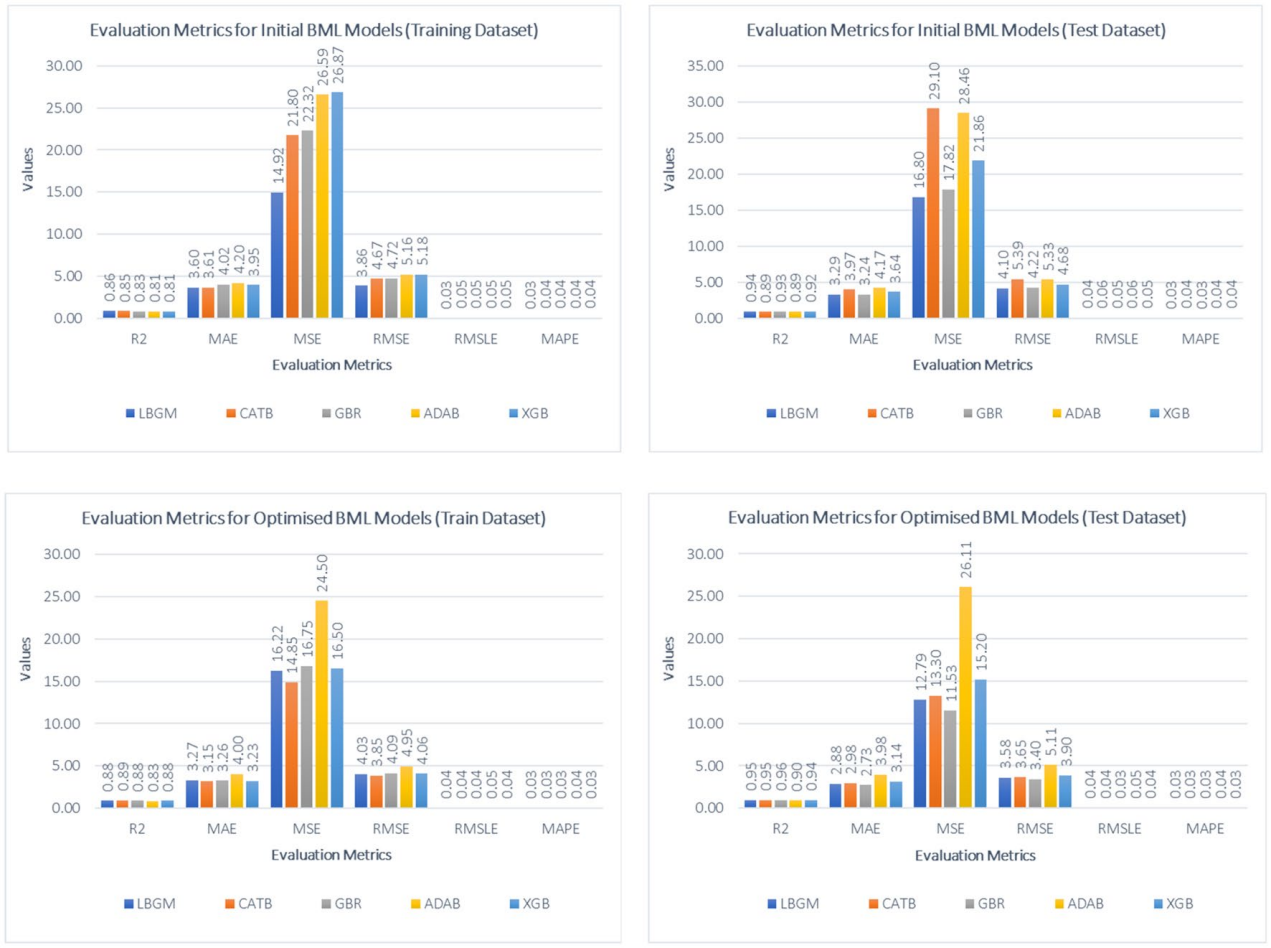
Comparison between BML and RS optimised models.

The comparison of the training and test datasets for both the initial and optimised BML models is shown in Fig. 8. In general, the RS algorithm improves prediction accuracy and reduces the modelling error for the training dataset of all BML models. However, the optimised ADAB model show a minor deficiency compared to the training results. The overall performance of BML models with RS optimisation shows that the GBR model is the best performing model with highest prediction accuracy and lowest modelling errors while the LGBM model are the best model without any optimisations with highest prediction accuracy and lowest modelling errors.

**Figure 9:**
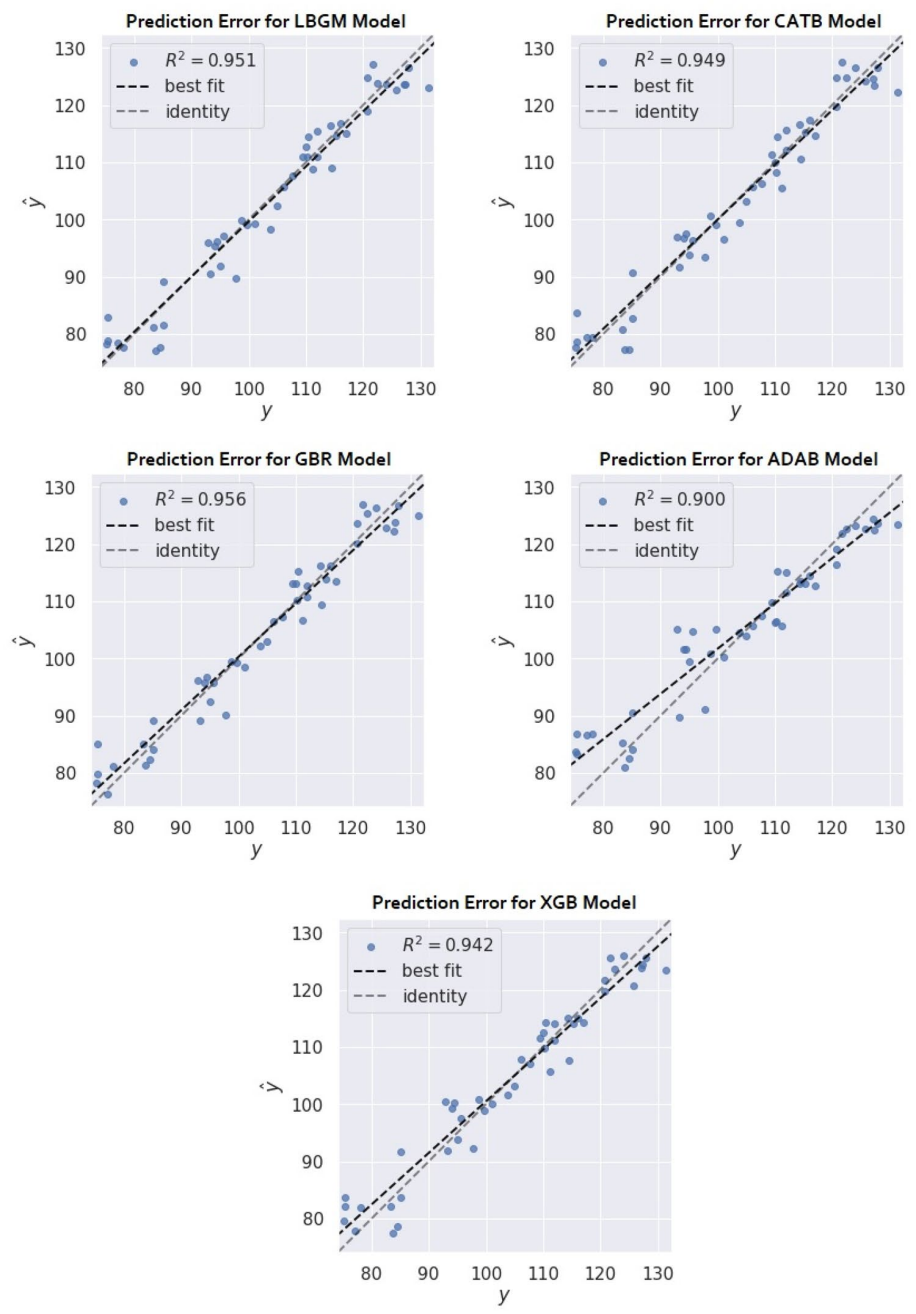
Best fit line for prediction distribution (RS model).

The prediction distributions of the optimised BML models appear to have a similar pattern for both the training and test datasets, with only a minor difference in prediction scores. The RS algorithms optimise the BML models to obtain a high prediction accuracy and a low error rate by tuning the hyperparameters for both the training and test datasets while simultaneously improving model performance. As presented in Fig. 9, the LBGM, CATB, and GBR all suggest a closed space between best fit and the identity line, demonstrating that the model's predictions are highly accurate.

### Features importance analysis

The explainability and interpretability of ML models are active areas of research that seek to understand why and how an ML model predicts output values. Numerous techniques, including feature importance analysis (FIA), are frequently used to explain and interpret ML models^68^. The permutation FIA techniques are model-dependent, which means they evaluate model predictions rather than the actual data. The explainability and interpretability metrics reveal how well ML model predictions correspond to physical knowledge. Additionally, it enables the discovery of hidden correlations between targets and features that are not readily visible in the data by allowing ML models to make correct predictions.

The original dataset is updated for each feature by randomly shuffling the feature values. The model's evaluation metric for the updated dataset is computed and compared to the original dataset's evaluation metric. This procedure is repeated numerous times for each feature to get the mean and standard deviation of the permutation importance score.

**Figure 10:**
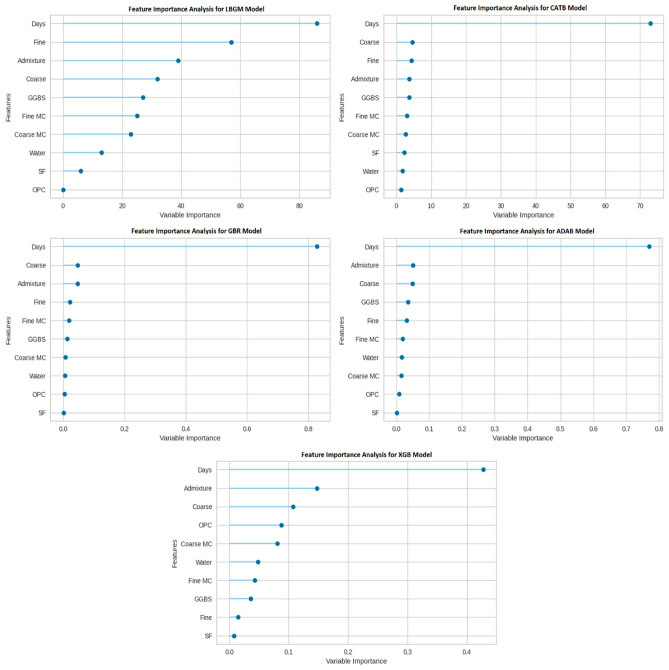
Feature importance analysis of BML models.

In this research, the permutation FIA was performed in all BML models to understand the influence of each feature/component of concrete in predicting the compressive strength of concrete. Figure 10 below displays all the features used in the compressive strength prediction model and their relative importance. ‘Days’ are an essential feature for all BML models, and SF is the least important feature in GBR, ADAB, and XGB models. It demonstrates that changes to the day's value in the dataset substantially affect the concrete compressive strength results. In contrast, changes in SF value have a considerably low impact on the strength prediction.

### Comparison between various ML algorithms

Subsequently, the initial BML models without optimisation was compared to 14 commonly used ML algorithms, including linear regression (LR), decision trees (DT), random forests (RF), and extra trees (ET), etc. Table 6 shows the summary of prediction accuracy and the evaluation metrics for 14 comparison ML models. For Table 6, only test dataset values were provided as the purpose of this section is to make comparison between BML model and other conventional models. Generally, all comparison models exhibit much lower prediction scores and more significant prediction errors than BML models. The comparison models show that the ET and RF models were the best performing model with an R^2^ of 0.78. Similarly, both models produced prediction errors, i.e., MAE and RMSE of 4.17 and 5.0, respectively. Overall, the comparison models demonstrate that the initial BML model outperforms all other machine learning models.

**Table 6:** Summary of comparison between various ML models.

Model	R^2^	MAE	MSE	RMSE	RMSLE	MAPE
Extra Trees	0.78	4.17	25.41	5.04	0.05	0.04
Random Forest	0.78	4.17	26.07	5.11	0.05	0.04
K Neighbours	0.72	4.90	35.09	5.92	0.05	0.05
Ridge	0.66	5.19	41.85	6.47	0.06	0.05
Least Angle	0.66	5.22	42.16	6.49	0.06	0.05
Linear	0.65	5.22	42.16	6.49	0.06	0.05
Elastic Net	0.65	5.41	45.79	6.77	0.06	0.05
Huber	0.64	5.30	44.55	6.67	0.06	0.05
Bayesian Ridge	0.64	5.54	47.42	6.89	0.07	0.05
Lasso	0.60	5.59	48.36	6.95	0.07	0.06
Decision Tree	0.59	5.59	45.66	6.76	0.07	0.05
Orthogonal Matching Pursuit	0.59	6.05	56.49	7.52	0.07	0.06
Passive Aggressive	0.25	7.80	96.13	9.80	0.09	0.08
Lasso Least Angle	0.09	9.84	153.55	12.39	0.12	0.10

## Conclusion and recommendation

Comparing all 5 BML models, the GBR model has outperformed the LBGM, CATB, ADAB, and XGB models. The GBR model optimised with RS algorithms achieved the highest prediction accuracy of 0.96 and the least prediction errors, with an MAE of 2.73, an RMSE of 3.40, and an RMSLE of 0.03. Notably, the RS algorithms optimisation technique improved the model prediction accuracy and reduced the modelling errors in all 5 BML models. Simultaneously, the evaluation of 14 commonly used ML models also suggests that the BML models have superior prediction accuracy and minimum prediction errors. These studies conclude that the optimised BML models, i.e., the GBR model are the best choice to predict the compressive strength of concrete, mainly for HPC and concrete with high volume GGBS replacements. For future research, a comparison study between ANN models with BML models or hyperparameter tuning with different optimisation algorithms, i.e., Grid Search, can be evaluated and compared with the proposed BML model's performance.

## Supplementary Information

Supplementary Information.

## Data Availability

All data generated or analysed during this study are included in this published article [and its supplementary information files]. GitHub: https://github.com/vilini007/HPC-GGBS-Concrete/blob/bae42e3d9f59743961176e43a4a504c51109e9c7/HPC_GGBS.ipynb. Google Colab: https://colab.research.google.com/drive/1fZzZfuTKI9MvD4enxCGiEVB0_d8p62Jk?usp=sharing.

## References

[1] Chung KL, Wang L, Ghannam M, Guan M, Luo J (2020). Prediction of concrete compressive strength based on early-age effective conductivity measurement. J. Build. Eng..

[2] Nguyen KT, Nguyen QD, Le TA, Shin J, Lee K (2020). Analyzing the compressive strength of green fly ash based geopolymer concrete using experiment and machine learning approaches. Constr. Build. Mater..

[3] Gomaa E, Han T, ElGawady M, Huang J, Kumar A (2021). Machine learning to predict properties of fresh and hardened alkali-activated concrete. Cement Concrete Composites.

[4] Chiew, F. H. Prediction of blast furnace slag concrete compressive strength using artificial neural networks and multiple regression analysis. *Proceedings - 2019 International Conference on Computer and Drone Applications, IConDA 2019*, pp. 54–58, 2019, 10.1109/IConDA47345.2019.9034920.

[5] Kang MC, Yoo DY, Gupta R (2021). Machine learning-based prediction for compressive and flexural strengths of steel fiber-reinforced concrete. Constr. Build. Mater..

[6] Han T, Siddique A, Khayat K, Huang J, Kumar A (2020). An ensemble machine learning approach for prediction and optimization of modulus of elasticity of recycled aggregate concrete. Constr. Build. Mater..

[7] Singh, P., Khaskil, P. Prediction of compressive strength of green concrete with admixtures using neural networks. *2020 IEEE International Conference on Computing, Power and Communication Technologies, GUCON 2020*, no. cm, pp. 714–717, 2020, 10.1109/GUCON48875.2020.9231230.

[8] Feng DC (2020). Machine learning-based compressive strength prediction for concrete: An adaptive boosting approach. Constr. Build. Mater..

[9] Mousavi SM, Aminian P, Gandomi AH, Alavi AH, Bolandi H (2012). A new predictive model for compressive strength of HPC using gene expression programming. Adv. Eng. Softw..

[10] Ben-Chaabene W, Flah M, Nehdi ML (2020). “Machine learning prediction of mechanical properties of concrete: Critical review. Constr. Build. Mater..

[11] Aliev K, Antonelli D (2021). Proposal of a monitoring system for collaborative robots to predict outages and to assess reliability factors exploiting machine learning. Appl. Sci. (Switzerland).

[12] Bahaghighat M, Abedini F, Xin Q, Zanjireh MM, Mirjalili S (2021). Using machine learning and computer vision to estimate the angular velocity of wind turbines in smart grids remotely. Energy Rep..

[13] Dangut MD, Skaf Z, Jennions IK (2021). An integrated machine learning model for aircraft components rare failure prognostics with log-based dataset. ISA Trans..

[14] Moshtaghzadeh M, Bakhtiari A, Izadpanahi E, Mardanpour P (2022). Artificial Neural Network for the prediction of fatigue life of a flexible foldable origami antenna with Kresling pattern. Thin-Walled Struct..

[15] Degtyarev VV, Naser MZ (2021). Boosting machines for predicting shear strength of CFS channels with staggered web perforations. Structures.

[16] Selvaraj S, Sivaraman S (2019). Prediction model for optimized self-compacting concrete with fly ash using response surface method based on fuzzy classification. Neural Comput. Appl..

[17] Castelli M, Vanneschi L, Silva S (2013). Prediction of high performance concrete strength using Genetic Programming with geometric semantic genetic operators. Expert Syst. Appl..

[18] Sun H, Burton HV, Huang H (2021). Machine learning applications for building structural design and performance assessment: State-of-the-art review. J. Build. Eng..

[19] Naranjo-Pérez J, Infantes M, Fernando Jiménez-Alonso J, Sáez A (2020). A collaborative machine learning-optimization algorithm to improve the finite element model updating of civil engineering structures. Eng. Struct..

[20] Abuodeh OR, Abdalla JA, Hawileh RA (2020). Assessment of compressive strength of Ultra-high Performance Concrete using deep machine learning techniques. Appl. Soft Comput. J..

[21] Khademi F, Jamal SM, Deshpande N, Londhe S (2016). Predicting strength of recycled aggregate concrete using Artificial Neural Network, Adaptive Neuro-Fuzzy Inference System and Multiple Linear Regression. Int. J. Sustain. Built Environ..

[22] Yan K, Shi C (2010). Prediction of elastic modulus of normal and high strength concrete by support vector machine. Constr. Build. Mater..

[23] Azimi-Pour M, Eskandari-Naddaf H, Pakzad A (2020). Linear and non-linear SVM prediction for fresh properties and compressive strength of high volume fly ash self-compacting concrete. Constr. Build. Mater..

[24] Seleemah AA (2012). A multilayer perceptron for predicting the ultimate shear strength of reinforced concrete beams. J. Civil Eng. Constr. Technol..

[25] Kaloop MR, Kumar D, Samui P, Hu JW, Kim D (2020). Compressive strength prediction of high-performance concrete using gradient tree boosting machine. Constr. Build. Mater..

[26] Lee S, Vo TP, Thai HT, Lee J, Patel V (2021). Strength prediction of concrete-filled steel tubular columns using Categorical Gradient Boosting algorithm. Eng. Struct..

[27] Aslam F (2020). Applications of gene expression programming for estimating compressive strength of high-strength concrete. Adv. Civil Eng..

[28] Lim CH, Yoon YS, Kim JH (2004). Genetic algorithm in mix proportioning of high-performance concrete. Cem. Concr. Res..

[29] Yeh IC (1998). Modeling of strength of high-performance concrete using artificial neural networks. Cem. Concr. Res..

[30] Milovancevic M, Denić N, Ćirković B, Nešić Z, Paunović M, Stojanović J (2021). Prediction of shear debonding strength of concrete structure with high-performance fiber reinforced concrete. Structures.

[31] Kaveh A, Bakhshpoori T, Hamze-Ziabari SM (2018). M5’ and mars based prediction models for properties of selfcompacting concrete containing fly ash. Periodica Polytechnica Civil Eng..

[32] Ahmad A (2021). Prediction of compressive strength of fly ash based concrete using individual and ensemble algorithm. Materials.

[33] Bui DK, Nguyen T, Chou JS, Nguyen-Xuan H, Ngo TD (2018). A modified firefly algorithm-artificial neural network expert system for predicting compressive and tensile strength of high-performance concrete. Constr. Build. Mater..

[34] Sargam Y, Wang K, Cho IH (2020). Machine learning based prediction model for thermal conductivity of concrete. J. Build. Eng..

[35] Cai R (2020). Prediction of surface chloride concentration of marine concrete using ensemble machine learning. Cement Concrete Res..

[36] Asteris PG, Kolovos KG, Douvika MG, Roinos K (2016). Prediction of self-compacting concrete strength using artificial neural networks. Eur. J. Environ. Civ. Eng..

[37] Siddique R, Aggarwal P, Aggarwal Y (2011). Prediction of compressive strength of self-compacting concrete containing bottom ash using artificial neural networks. Adv. Eng. Softw..

[38] Słoński M (2010). A comparison of model selection methods for compressive strength prediction of high-performance concrete using neural networks. Comput. Struct..

[39] Lin SS, Shen SL, Zhou A, Xu YS (2021). Risk assessment and management of excavation system based on fuzzy set theory and machine learning methods. Autom. Constr..

[40] Xu H (2020). Identifying diseases that cause psychological trauma and social avoidance by GCN-Xgboost. BMC Bioinf..

[41] Dhananjay B, Sivaraman J (2021). Analysis and classification of heart rate using CatBoost feature ranking model. Biomed. Signal Process. Control.

[42] Kivrak M, Guldogan E, Colak C (2021). Prediction of death status on the course of treatment in SARS-COV-2 patients with deep learning and machine learning methods. Comput. Methods Programs Biomed..

[43] Farooq F, Ahmed W, Akbar A, Aslam F, Alyousef R (2021). Predictive modeling for sustainable high-performance concrete from industrial wastes: A comparison and optimization of models using ensemble learners. J. Clean. Prod..

[44] Balf FR, Kordkheili HM, Kordkheili AM (2021). A new method for predicting the ingredients of self-compacting concrete (SCC) including fly ash (FA) using data envelopment analysis (DEA). Arab. J. Sci. Eng..

[45] Nguyen-Sy T, Wakim J, To QD, Vu MN, Nguyen TD, Nguyen TT (2020). Predicting the compressive strength of concrete from its compositions and age using the extreme gradient boosting method. Constr. Build. Mater..

[46] Asteris PG, Kolovos KG (2019). Self-compacting concrete strength prediction using surrogate models. Neural Comput. Appl..

[47] Zhang J, Ma G, Huang Y, Sun J, Aslani F, Nener B (2019). Modelling uniaxial compressive strength of lightweight self-compacting concrete using random forest regression. Constr. Build. Mater..

[48] Yu Y, Li W, Li J, Nguyen TN (2018). A novel optimised self-learning method for compressive strength prediction of high performance concrete. Constr. Build. Mater..

[49] Goliatt, L. & Farage, M. R. C. An extreme learning machine with feature selection for estimating mechanical properties of lightweight aggregate concretes. *2018 IEEE congress on evolutionary computation, CEC 2018 - Proceedings*, 2018, 10.1109/CEC.2018.8477673.

[50] Ke, G. *et al.*, “LightGBM: A highly efficient gradient boosting decision tree. *Adv. Neural Inf. Process. Syst.*, vol. 2017-Decem, no. Nips, pp. 3147–3155 (2017).

[51] Hancock JT, Khoshgoftaar TM (2020). CatBoost for big data: An interdisciplinary review. J. Big Data.

[52] Prokhorenkova L, Gusev G, Vorobev A, Dorogush AV, Gulin A (2018). Catboost: Unbiased boosting with categorical features. Adv. Neural Inf. Process. Syst..

[53] Bakouregui AS, Mohamed HM, Yahia A, Benmokrane B (2021). Explainable extreme gradient boosting tree-based prediction of load-carrying capacity of FRP-RC columns. Eng. Struct..

[54] Gong M, Bai Y, Qin J, Wang J, Yang P, Wang S (2020). Gradient boosting machine for predicting return temperature of district heating system: A case study for residential buildings in Tianjin. J. Build. Eng..

[55] Pham BT (2021). A novel approach for classification of soils based on laboratory tests using Adaboost, Tree and ANN modeling. Transp. Geotech..

[56] Liu Q, Wang X, Huang X, Yin X (2020). Prediction model of rock mass class using classification and regression tree integrated AdaBoost algorithm based on TBM driving data. Tunnell. Undergr. Space Technol..

[57] Tang, Q., Xia, G., Zhang, X., & Long, F. A customer churn prediction model based on XGBoost and MLP. *Proceedings - 2020 International Conference on Computer Engineering and Application, ICCEA 2020*, pp. 608–612, 2020, 10.1109/ICCEA50009.2020.00133.

[58] Mustika, W. F., Murfi, H., Widyaningsih, Y. Analysis accuracy of XGBoost model for multiclass classification - a case study of applicant level risk prediction for life insurance,” *Proceeding - 2019 5th International Conference on Science in Information Technology: Embracing Industry 4.0: Towards Innovation in Cyber Physical System, ICSITech 2019*, pp. 71–77, 2019, 10.1109/ICSITech46713.2019.8987474.

[59] Wu, D., Guo, P., Wang, P. Malware Detection based on Cascading XGBoost and Cost Sensitive. *Proceedings - 2020 International Conference on Computer Communication and Network Security, CCNS 2020*, pp. 201–205, 2020, 10.1109/CCNS50731.2020.00051.

[60] Li, J. & Zhang, R. Dynamic weighting multi factor stock selection strategy based on XGboost machine learning algorithm. *Proceedings of 2018 IEEE international conference of safety produce informatization, IICSPI 2018*, pp. 868–872, 2019, 10.1109/IICSPI.2018.8690416.

[61] Qu Y, Lin Z, Li H, Zhang X (2019). Feature recognition of urban road traffic accidents based on GA-XGBoost in the context of big data. IEEE Access.

[62] Chen T, Guestrin C (2016). XGBoost: A scalable tree boosting system. Proc. ACM SIGKDD Int. Conf. Knowl. Discov. Data. Min..

[63] “Welcome to PyCaret - PyCaret Official.” https://pycaret.gitbook.io/docs/ (accessed Apr. 25, 2022).

[64] Lasisi, A., Sadiq, M. O., Balogun, I., Tunde-Lawal, A., & Attoh-Okine, N. A boosted tree machine learning alternative to predictive evaluation of nondestructive concrete compressive strength. *Proceedings - 18th IEEE International Conference on Machine Learning and Applications, ICMLA 2019*, pp. 321–324, 2019, 10.1109/ICMLA.2019.00060.

[65] Nguyen H, Vu T, Vo TP, Thai HT (2021). Efficient machine learning models for prediction of concrete strengths. Constr. Build. Mater..

[66] Sen Fan, R., Li, Y., Ma, T. T. Research and Application of Project Settlement Overdue Prediction Based on XGBOOST Intelligent Algorithm. *iSPEC 2019 - 2019 IEEE Sustainable Power and Energy Conference: Grid Modernization for Energy Revolution, Proceedings*, pp. 1213–1216, 2019, 10.1109/iSPEC48194.2019.8975056.

[67] Bergstra J, Bengio Y (2012). Random search for hyper-parameter optimization. J. Mach. Learn. Res..

[68] Pan Y, Zhang L (2020). Data-driven estimation of building energy consumption with multi-source heterogeneous data. Appl. Energy.

